# NeuroMArVL: An interactive and collaborative web-based tool for visualizing brain networks

**DOI:** 10.1162/NETN.a.569

**Published:** 2026-07-20

**Authors:** Christopher Leslie Adamson, Mehul Gajwani, Matthias Klapperstueck, James Manley, Tim Dwyer, Alex Fornito

**Affiliations:** Turner Institute for Brain and Mental Health, School of Psychological Sciences, and Monash Biomedical Imaging, Monash University, Melbourne, Australia; Faculty of Information Technology, Monash University, Melbourne, Australia

**Keywords:** connectome, visualization, web, software

## Abstract

Brain connectivity data are high-dimensional and are often modeled as graphs comprising in the order of ∼10^2^–10^4^ nodes connected by around 10^3^–10^6^ edges. Generating useful visualizations is essential for reducing and understanding such complexity. Indeed, this complexity offers a particular challenge for transparent science, since investigators must often choose a specific snapshot of a visualization for publication that often overlooks much of the rich detail present in the data. A further challenge for neuroscience is that brains are physical systems, and it is often important to consider how topological properties of the connectome, which can be visualized within arbitrarily abstract spaces, relate to their physical embedding. Most available tools offer visualizations for physically or topologically embedded representations without a clear mapping between the two. Here, we introduce NeuroMArVL, a novel, open-source, web-based brain connectome visualization tool that offers numerous features for moving seamlessly between, and interacting with, different physical and topological representations of connectome data. Critically, visualization data and parameters can be saved locally or on the web server as shareable links, facilitating reuse, collaboration, and open, transparent reporting of results in publications. The software can be freely accessed at https://immersive.erc.monash.edu/neuromarvl/.

## INTRODUCTION

Graph theory has become a popular tool for flexibly mapping and modeling brain networks at various scales, from individual neurons to large brain regions (Bullmore et al., 2009; Fornito et al., 2016; Sporns et al., 2005). It is especially common in human neuroimaging analysis, where graphs represent macroscopic areas and edges reflect structural connectivity (SC) or functional coupling (FC). This modeling treats the brain as part of a broader class of complex networks (Bullmore et al., 2009; Fornito et al., 2016), making it accessible to diverse analytic tools from math, physics, and other sciences (Albert & Barabási, 2002; Newman, 2003).

Data visualization is essential in these analyses, as the resulting networks are often topologically complex and high-dimensional. Even at macroscopic neuroimaging resolutions, networks usually contain around 10^2^–10^3^ nodes and 10^3^–10^5^ edges. To manage this complexity, several visualization strategies have been developed. Common neuroscience approaches fall into three main categories (Chopra et al., 2023; Margulies et al., 2013): anatomical, circular, and topological—each with distinct strengths and limitations.

Anatomical projections place nodes based on their physical locations in a 3D brain coordinate system. They are useful for localizing specific brain effects, but projecting 3D networks into 2D figures for publication can lose significant information and obscure higher-order topological features.

Circular projections place nodes around a circle’s perimeter, with edges as straight lines or curves through the interior. Varying node order can highlight different network properties; for instance, ordering by network or community assignments reveals modular organization (e.g., Fulcher & Fornito, 2016; Van Horn et al., 2012). Additional features can be shown using glyphs or bars arranged concentrically around the nodes (e.g., Fulcher & Fornito, 2016; Irimia et al., 2012; Van Horn et al., 2012).

Topological projections disregard anatomical or geometric constraints, positioning nodes based on their topological relationships. Examples include multidimensional scaling, which aligns projections with edge distances (Dwyer et al., 2006; Kruskal, 1964), and force-directed methods, which treat the graph as a system of nodes influenced by attractive or repulsive forces tied to properties like edge weights or shortest path lengths (Fruchterman & Reingold, 1991; Kamada & Kawai, 1989). Node layouts are usually optimized by functions minimizing edge crossings and promoting symmetric, spatially uniform distributions (Gibson et al., 2013).

There are many software packages that can be used to generate topological and circular projections in both neuroscience and the broader field of network science. For instance, NetworkX (https://networkx.org), GraphViz (https://graphviz.org/), and Gephi (https://gephi.org/) are popular tools for visualizing networks derived from any kind of data and offer a range of distinct types of circular and topological projections. Circos (https://circos.ca) is a tool that specializes in circular graph representations for genetic data.

Anatomical projections can generally only be generated using tools developed specifically for the neuroscientific context. These include BrainNet Viewer (Xia et al., 2013), NetPlotBrain (https://www.netplotbrain.org/), Brainconn (https://github.com/sidchop/brainconn), Graphpype (https://neuropycon.github.io/graphpype/index.html), and Nilearn (https://github.com/nilearn/nilearn), which allow plotting of networks overlaid onto template brainlike surfaces.

These tools enable diverse brain network visualizations but have key limitations. Many are noninteractive, producing static images that cannot be rotated, zoomed, or explored at the level of individual nodes and edges—crucial for understanding 3D brain structures. Interactive platforms would let users query node and edge properties directly from the visualization, avoiding reliance on raw data. Also, current tools do not support yoked visualizations across different projection types, making it hard to track nodes across anatomical and topological views. Lastly, sharing visualizations is limited; collaborators must exchange static images or raw data and code. Interactive sharing would enhance collaboration, reproducibility, and transparency in publications.

To meet these needs, we introduce NeuroMArVL, a free, web-based tool for creating and sharing interactive brain network visualizations. It provides fully interactive 2D and 3D views of anatomical, circular, and topological projections, including yoked^1^ visualizations that link different network representations. This concurrent viewing enables seamless switching between projections, enhancing data interpretation. Users can also generate a unique URL for any visualization, making it easy to share or embed in scientific manuscripts. We describe the tool’s features here; it is available at https://immersive.erc.monash.edu/NeuroMArVL/, with code at https://github.com/NSBLab/NeuroMArVL.

## IMPLEMENTATION DETAILS

NeuroMArVL was developed for use in a web browser using TypeScript and JavaScript. This development environment was chosen for deployment to a webserver. Connectomes are defined using vertices or nodes (regions of interest) and edges (connections between nodes) and are visualized using different types of ball-and-stick diagrams.

### Webpage Components

The layout of the webpage components and widgets is managed using Bootstrap 3 (https://getbootstrap.com). Bootstrap is used to manage the layout and interactivity of NeuroMArVL. The overall layout of the site, including the locations of panels, buttons, and viewings windows, is rendered using Bootstrap. Interactive elements, including dropdown menus, buttons, sliders, and file-selection buttons, are also rendered using Bootstrap.

### 3D Visualizations

3D objects, such as surface meshes and ball-and-stick sprites, are rendered and manipulated using three.js version 99 (https://threejs.org). Three.js is a lightweight, cross-browser, general-purpose 3D library commonly used to render objects, animate objects, and create scenes. In NeuroMArVL, three.js is used to render the anatomical projection, that is, the brain surface, nodes, and edges. This includes the surface mesh and the ball-and-stick model used to show the node locations and edge strengths. Three.js allows customization of many features of these objects, including the color and opacity of the surface, the size and color of the nodes, and the thickness and color of the edges. This functionality is made accessible to the user, with several options for manipulating the appearance of the projections (see Usage Details).

NeuroMArVL is also optionally able to render a 3D nonanatomical visualization using the Constrained Layout (CoLa) algorithm (Dwyer et al., 2006). This algorithm extends force-directed visualizations by adding separation constraints, which enforce a minimum distance between pairs of nodes (Dwyer et al., 2006). The additional separation constraints facilitate the layout of graphs with nonoverlapping node labels and in specific modules/clusters. As above, the ball-and-stick model generated by CoLa is also rendered using three.js.

NeuroMArVL also supports yoking between the two visualizations. Yoking allows for selection of a node on one visualization to automatically highlight the corresponding node (and its edges) on the other visualization.

### 2D Visualizations

NeuroMArVL provides options to generate different 2D projections of the connectivity data. These visualizations may highlight connectivity patterns such as modular community structure or rich-club connectivity patterns that may not be clear on the 3D visualization. Besides the 3D implementation of the CoLA algorithm above, NeuroMArVL also supports 2D visualization using the same algorithm and implementation.

NeuroMArVL also allows users to create 2D nonanatomical projections using the Compound Spring Embedder (COSE) layout algorithm (Dogrusoz et al., 2009). The COSE algorithm is a physics-based method that produces fast results for large, complex graphs. It models the interactions between nodes using several physical relationships: “electric charge” attraction/repulsion between nodes; “springs” of a prespecified desired length; “gravitational forces” acting to keep graph components together; and “minor repulsion forces” that prevent node-to-node overlaps. NeuroMArVL also supports the COSE-bilkent algorithm, a modification of the COSE algorithm that is optimized for larger graphs and can perform multilevel nesting of nodes into clusters (Dogrusoz et al., 2009).

Circular plots are generated using custom code using the cluster layout in d3.js. The nodes are placed around the edge of the circle with connecting arcs denoting edges of the adjacency/weight matrices. A hierarchical bundling can be applied to group nodes by a chosen attribute, such as a module identifier. When bundling is used, interbundle arcs can be distinguished from intrabundle arcs.

These visualizations are implemented using a combination of two JavaScript libraries. Firstly, Cytoscape.js 2.7.2 (Shannon et al., 2003) implements the CoLA, COSE, and COSE-bilkent layout algorithms. Cytoscape.js is a JavaScript library for visualization and analysis; in NeuroMArVL, it is used to compute the graph layouts based on the user-supplied connectivity information. Then, D3.js v3 (https://d3js.org) is used to render the visualizations. D3.js is a library used to create dynamic and interactive 2D visualizations; here, it is used to render the graph layouts generated by Cytoscape.js, resulting in interactive 2D objects that can be panned and zoomed. As with the above 3D visualizations, the 2D visualizations are also yoked.

### Included Data

NeuroMArVL comes inbuilt with several sets of inbuilt and example data (Table 1), particularly for human cortex. These include various human cortical surface templates, surfaces extracted from volumetric masks of other structures, example connectomes, network allocations according to common network schemes, and example attributes. These have been chosen from a representative set of commonly used parcellations. The cortical surfaces are provided as fixed options in the NeuroMArVL web interface, while the connectomes and other data can be easily downloaded from the repository and used as desired.

**Table 1.** Example datasets formatted to be compatible with NeuroMArVL

Structure	Data	Name, resolution	References	Example URL	Additional information
Human cortex	Surface	ICBM152	(Mazziotta et al., 2001)	https://immersive.erc.monash.edu/neuromarvl/?example=e4e59658-c7c6–4092-b19c-14fbde2dd14b_58.178.236.210	– Sourced from BrainNet Viewer (Xia et al., 2013)
		Ch2	(Holmes et al., 1998)	https://immersive.erc.monash.edu/neuromarvl/?save=43e058c7-e7d7-4aa8-b950-55c63e1e0d84_49.127.87.145	
		fsaverage, 32 k	(Fischl et al., 1999)	https://immersive.erc.monash.edu/neuromarvl/?save=4965ef7a-1515-41a9–8647-68a557caf273_49.127.87.145	– Sourced from neuromaps (Markello et al., 2022)
		fsLR, 41 k	(Van Essen et al., 2012)	https://immersive.erc.monash.edu/neuromarvl/?save=4be6c4e7-590b-4cef-a957-21f71bf63007_49.127.87.145	– Inflated, spherical, and midthickness/pial/white matter surfaces available for each
		CIVET, 41 k	(Fonov et al., 2009)	https://immersive.erc.monash.edu/neuromarvl/?save=ea6c2db2-4df1-4657-849c-9e04c9466d72_49.127.87.145	
	Nodes Attributes	Desikan-Killiany, 68 nodes	(Desikan et al., 2006)	https://immersive.erc.monash.edu/neuromarvl/?save=5e44af19-821a-461c-a520-9ceeb8cebdb6_49.127.87.145	– Attributes include anatomical lobe
	Labels	HCP-MMP1, 360 nodes	(Glasser et al., 2016)	https://immersive.erc.monash.edu/neuromarvl/?save=34038bb7-afc8–4739-a2b3-962b612e62d8_49.127.87.145	– Attributes include network according to Cole-Anticevic scheme (Ji et al., 2019)
	Connectome^a^	Schaefer, 100–900 nodes	(Schaefer et al., 2018)	https://immersive.erc.monash.edu/neuromarvl/?save=2af0b5cd-942d-463f-893c-1c98ba5d310a_49.127.87.145	– Attributes include network allocation according to Yeo 7- and 17-network schemes (Yeo et al., 2011)
Human subcortex	Surface	Melbourne Subcortical Atlas, 16–54 nodes	(Oldham et al., 2020; Tian et al., 2020)	https://immersive.erc.monash.edu/neuromarvl/?save=55fe03f4-f2b3-4fd8-9993-f94157774a88_49.127.87.145	– Surfaces extracted from volume
	Nodes				– Nodes extracted as the center of each subcortical area
	Connectome				– Connectome derived from diffusion MRI
	Attributes				
	Labels				
Human brainstem	Surface	67 nodes	(Hansen et al., 2024)	https://immersive.erc.monash.edu/neuromarvl/?save = 23d3a5a1–5210-4803-b459-aa699a91f78b_49.127.87.145	– Surfaces extracted from volume
	Nodes				– Connectome derived from functional MRI
	Connectome				
	Attributes				
	Labels				
Mouse whole brain	Surface	112 nodes	(Rubinov et al., 2015)	https://immersive.erc.monash.edu/neuromarvl/?save=b15a81f1-9be2-43c4-b46d-696bd0402871_49.127.87.145	– Registered to the Allen Common Coordinate Framework v3 (Wang et al., 2020)
	Nodes		https://github.com/netneurolab/netneurotools		– Surface extracted from volume
	Connectome				– Connectome derived from tract-tracing
	Attributes				
	Labels				

These datasets are included with the NeuroMArVL viewer or are available for download from the GitHub repository. Users are not restricted to these data and can input any data of their choosing.

^a^ Connectomes derived from diffusion MRI (Gajwani et al., 2023; Oldham et al., 2020).

The cortical surfaces are sourced from the BrainNet Viewer (Xia et al., 2013) and the neuromaps (Markello et al., 2022) packages (Figure 2C). From BrainNet Viewer, we use the ICBM152 surface (Mazziotta et al., 2001) and Ch2 (Colin27) surfaces (whole brain/cortex only) (Holmes et al., 1998). From neuromaps, we use fsaverage surfaces (Fischl et al., 1999), fsLR surfaces (Van Essen et al., 2012), and CIVET surfaces (Fonov et al., 2009); these include spherical, inflated, pial, midthickness, and white matter surfaces (as available from the toolbox). We also provide some additional example surfaces directly derived from volumetric data. These surfaces have not undergone additional preprocessing (such as smoothing) but are registered to common neuroanatomical spaces (MNI for human and Allen Brain Institute Common Coordinate Framework v3 (Allen CCF v3) for mouse) and can be used for visualization. These include surface renderings of human subcortical structures (namely, thalamus, hippocampus, amygdala, and globus pallidus) (Oldham et al., 2020; Tian et al., 2020), human brainstem (Hansen et al., 2024), and mouse whole-cortex surfaces (Rubinov et al., 2015).

Alongside these surfaces, we also include a suite of example data that can be used to generate visualizations (see Table 1 for a summary). These data include all the necessary coordinates, attributes, and labels required to generate the visualizations seen in all the figures in this manuscript. Specifically, we include example connectomes for human cortex in several parcellations: the Desikan-Killiany parcellation with 68 nodes (Desikan et al., 2006), the Human Connectome Project Multimodal Parcellation (HCP-MMP1) with 360 nodes (Glasser et al., 2016), and the Schaefer et al. parcellations with 100–900 nodes (Schaefer et al., 2018). We also include example data for human subcortex (all four resolutions of the Melbourne Subcortical Atlas) (Tian et al., 2020), human brainstem (Hansen et al., 2024), and mouse brain (Rubinov et al., 2015). These datasets comprise connectomes derived from the different modalities: The mouse connectome is derived from tract tracing, the brainstem connectome from fMRI, and the other connectomes from diffusion MRI (full processing details are available in the references associated with each paper). These data have all been formatted to the required specification and can be easily accessed via the repository associated with the NeuroMArVL viewer.

If users are working with connectomes parcellated according to any of the above schemes, using the NeuroMArVL viewer is extremely simple. Users can choose the brain surface for visualization, load the node coordinates/attributes/labels from the data contained in the repository, and then they need to provide only their connectome for visualization. If using a different parcellation scheme, users need only input the node coordinates and attributes alongside their connectome. Full usage details are provided in Usage Details.

### Saved Data

The hosted NeuroMARVL instance at https://immersive.erc.monash.edu/neuromarvl/ is located at the Australian Research Data Commons (ARDC) Nectar Research Cloud in Australia. The webserver is running on a two-account secured Windows Data Centre 2016 installation.

NeuroMArVL allows users to share data via links (see Reproducibility and Data sharing), which involves hosting uploaded data—such as connectomes, node locations, adjacency matrices, labels, attributes and custom brain models—in plaintext files within a single server directory. Each file is assigned a UUID-like random name to avoid identifiable information and file name collisions. While this makes unauthorized identification difficult, file contents are not encrypted. As links may be embedded in research outputs, data are hosted permanently and links do not expire. All data transfers occur via HTTPS through a web browser.

## USAGE DETAILS

The NeuroMArVL connectome visualization tool can be accessed online (https://immersive.erc.monash.edu/NeuroMArVL/) or can be built from the source code (https://github.com/NSBLab/NeuroMArVL) and deployed locally using Visual Studio. Google Chrome is the only browser currently supported.

NeuroMarvl is ideally suited to visualization of networks that can be embedded in Euclidean space (in three dimensions or fewer). Networks can have any number of nodes, but the maximum number of displayed edges is also set to 1,000 to facilitate efficient performance and to minimize visual clutter. NeuroMArVL comes with documentation built into the tool, which can be accessed by hovering the mouse cursor of each menu option. Several example datasets demonstrating the functionality of the tool can also be accessed (https://github.com/NSBLab/neuromarvl/blob/master/example_data).

**Figure 1.**
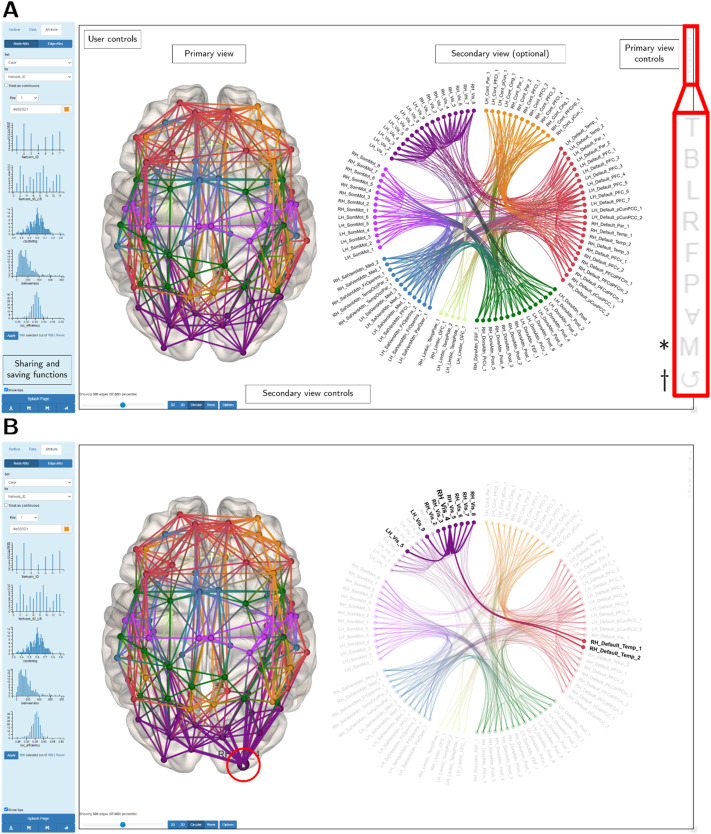
NeuroMArVL functionality. (A) An example connectome parcellated according to the Schaefer et al. 100-node parcellation (Schaefer et al., 2018) and colored according to the Yeo et al. 7-network scheme is shown (Yeo et al., 2011). NeuroMArVL can visualize connectomes on brain surfaces in 3D and generate accompanying 2D graph-based visualizations. Inset shows the controls for manipulating the “Primary” (3D) visualization (see also Interactively Adjusting Display Properties of the Network). (*) The split-brain view allows for visualization of the left and right hemispheres separately (see also Figure 3C); (†) Auto-rotation causes the primary view to rotate in an anticlockwise fashion according to the current view. Details of the blue user controls are explained in Figure 2. (B) An example of the yoking functionality, where nodes can be highlighted in the 3D and 2D visualization simultaneously. The highlighted node (see red circle; circle is not shown in software) is enlarged on the 3D visualization; the highlighted node, its neighbors, and their connecting edges are emboldened in the 2D visualization. The data and visualization in this figure can also be viewed interactively at https://immersive.erc.monash.edu/neuromarvl/?save=39bba064-915f-49d4-a25f-19e135e89747_49.127.51.226.

The primary display is split into a “User Controls” panel and visualization panel (Figure 1A). The user controls provide multiple options for inputting and visualizing data, spread across three main tabs. The *Surface* tab allows users to choose the specific brain surface they wish to use as an underlay for anatomical projections of the network data. Currently included options are summarized in Table 1. The tab also allows users to adjust the opacity of the surface visualization.

The *Data* tab allows users to specify the input data and the *Attributes* tab allows users to adjust various display properties. The available options for each of these tabs is discussed in the following sections.

### Data Inputs

**Figure 2.**
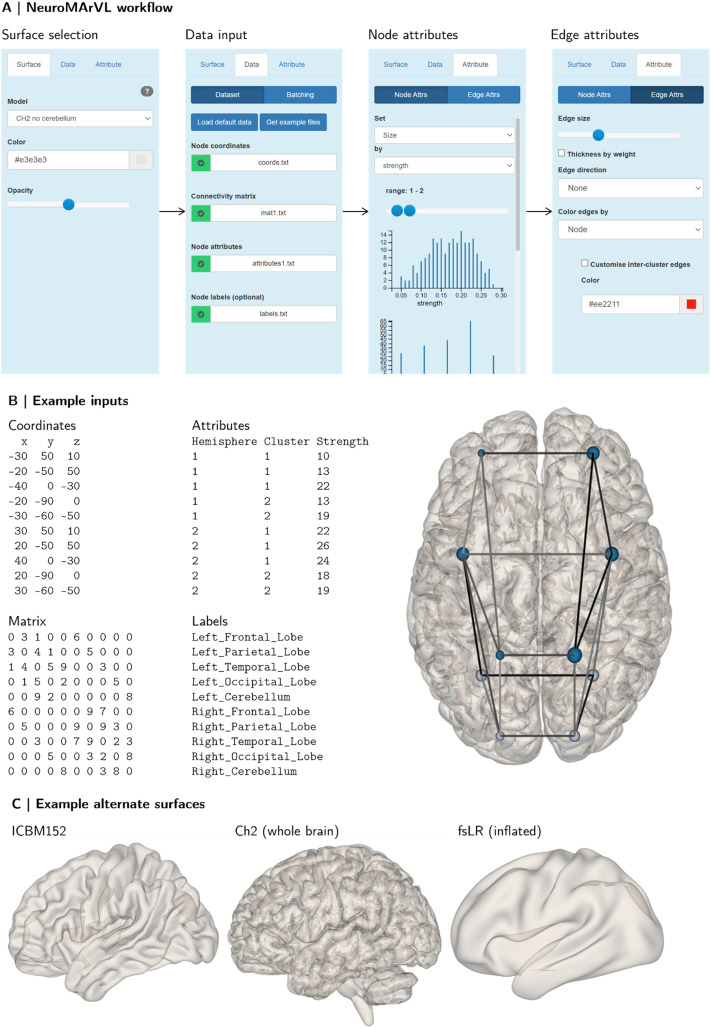
Data inputs and visualization in NeuroMArVL. (A) Workflow specifying properties of the primary view brain surface. First, users choose the brain model for visualization using the “Surface” tab. Second, input data are provided in the form of white space-delimited ASCII text files describing network connectivity and other properties using the “Data” tab. Third, the “Attributes” tab can be used to adjust the visualization of node and edge attributes. The “Node Attrs” subtab allows node properties such as size and color to be modified adaptively. Distributions below show the range of values for each property listed in the node attributes file. Select portions of each distribution can be interactively highlighted to reveal the corresponding nodes and edges in the graph visualizations. Finally, edge properties such as size, color, thickness, and representation of directionality can be modified using the “Edge Attrs” tab. (B) Example input data and corresponding visualization for a simple network. Top left shows the contents of the coordinates file, which locates the nodes for the visualization. Bottom left shows the contents of the matrix file, which encodes the connection strength between nodes. Top right shows the contents of the attributes file, which defines node-level properties. Bottom right shows the contents of the labels file (an optional input), which defines the name of each node. Far right shows the corresponding anatomical projection of the network using the CH2 surface. In this visualization, node size is given by strength (sum of edge weights at that node), node color by the cluster ID, and edge color by edge weight. The data and visualization in this panel can also be viewed interactively at https://immersive.erc.monash.edu/neuromarvl/?save=19df82e9-7bdf-4de6-824f-948e2a00d74a_49.127.37.54. (C) Visualization of brain surface models available in NeuroMArVL. Left, International Consortium for Brain Mapping (ICBM) 152-person template (cortical surface only); middle, Ch2 (whole brain); right, fsLR (inflated). Other surfaces are inbuilt in the viewer, including the Ch2 cortex only, fsLR 32 k surfaces, fsaverage 41k surfaces, and CIVET 41 k surfaces. Further surfaces have been formatted and made available for download from the repository, including surfaces for the human subcortex, human brainstem, and mouse whole brain.

NeuroMArVL requires three user-input files to generate a brain visualization, along with an additional two optional inputs, which are added using the page’s ‘Data’ tab (Figure 2A–B). The required data inputs are the spatial coordinates of each node, the adjacency/weight matrix encoding network connectivity, and attributes related to each node. The optional inputs are used to supply text labels/names for each node and to change the surface for the physical representation. Examples of the formats for each of the input files can be obtained from the GitHub repository, or can be downloaded from https://immersive.erc.monash.edu/neuromarvl/brain-app/data/example.zip.

The first required input is a *node coordinate file*, which should be specified in a whitespace (i.e., tab or space)-delimited ASCII text file containing *n* rows (where *n* is the number of nodes). Each row must contain three entries corresponding to the {*x*, *y*, *z*} coordinates of each node (Figure 2). For example, a volumetric parcellation could use each region’s center of gravity as its node coordinate. The node coordinates should be in register with the surface that is used for visualization, such as MNI152 coordinates for humans (which is otherwise used by default in NeuroMArVL) or the Allen CCF v3 for mice (for which an example surface is also provided) (see Table 1).

The second required input is an *adjacency* or *weight matrix*, which must be a whitespace-delimited text file with *n* × *n* noninfinite nonmissing entries, where each entry is the connectivity strength between regions (Figure 2A–B). Negative values are permitted, and a value of zero indicates no connection. The matrix may encode measures of SC (e.g., given by streamline counts estimated using diffusion magnetic resonance imaging, MRI), FC (e.g., given by correlations in functional MRI signal fluctuations), or any other directed or undirected similarity measure between nodes.

The final required input is an *attribute file*, which contains information about node-level features to be included in the visualization. This should be a whitespace-delimited file with *n* + 1 rows and *m* columns, where *m* is the number of attributes. The first row should be the names of the attributes, and the *n* rows below should contain the attribute values for each of the *n* regions (Figure 2A–B). Each attribute can be a binary, integer, or continuous scalar variable. Attributes may include features like node strength, node centrality estimates (e.g., betweenness centrality) or anatomical or functional features (e.g., region volume). Nodes may also be allocated to modules or communities (e.g., Newman, 2012) in the attributes file. NeuroMArVL allows nodes to be assigned to more than one module, where multiple affiliations are denoted using binary membership assignments separated by pipe delimiters. For instance, the vector [0 | 1 | 0 | 1] would indicate that a node belongs to Modules 2 and 4 of a four-module decomposition.

The first optional input is a *node label* file, corresponding to a single-column text file containing *n* node labels, represented as strings, that are used to annotate the visualizations (Figure 2B).

The second optional input allows users to specify a *custom surface* file, should those already supplied not be appropriate (Figure 2C). This custom surface must be supplied as a text .obj file containing *v* rows with the {*x, y, z*} coordinates of each vertex (preceded by the letter “v”), followed by *f* rows of 3-tuples that define each triangular face (preceded by the letter “f”). This format is similar to how a VTK polygon file is stored or how a GIfTI file is encoded.

### Interactively Adjusting Display Properties of the Network

Display properties of the loaded network can be interactively adjusted using the *Attributes* tab and additional buttons in the display window. The attributes tab contains two subtabs, *Node Attrs* and *Edge Attrs*, which allow users to adjust display properties of node and edge attributes, respectively.

#### Node attributes.

The sizes and colors of nodes can be adjusted using interactive sliders and color palettes. Node sizes can be adjusted using any of the node attributes loaded into the “Attributes” file. Each attribute will be named according to the column heading in that file.

The *Node Attrs* subtab additional displays the distribution of each loaded attribute. Users can click on this distribution to select specific ranges of values. The network visualization is then automatically updated to selectively highlight the nodes within the selected range. This feature allows users to interactively select subsets of nodes for visualization based on, for example, their values on a particular centrality measure or membership of a specific module.

#### Edge attributes.

In this subtab, users can globally scale the size of each edge or scale edge thicknesses according to their weight, as encoded in the *Connectivity matrix* input file. These weights can also be used to specific the color of each edge. Alternatively, edge colors can be assigned based on the nodes to they are attached. This option is particularly useful for coloring edges according to node module memberships. In this case, users can assign the color of each module and edge colors will be updated accordingly (intermodule edges are colored as a blend of their respect node colors by default, but can also be customized).

**Figure 3.**
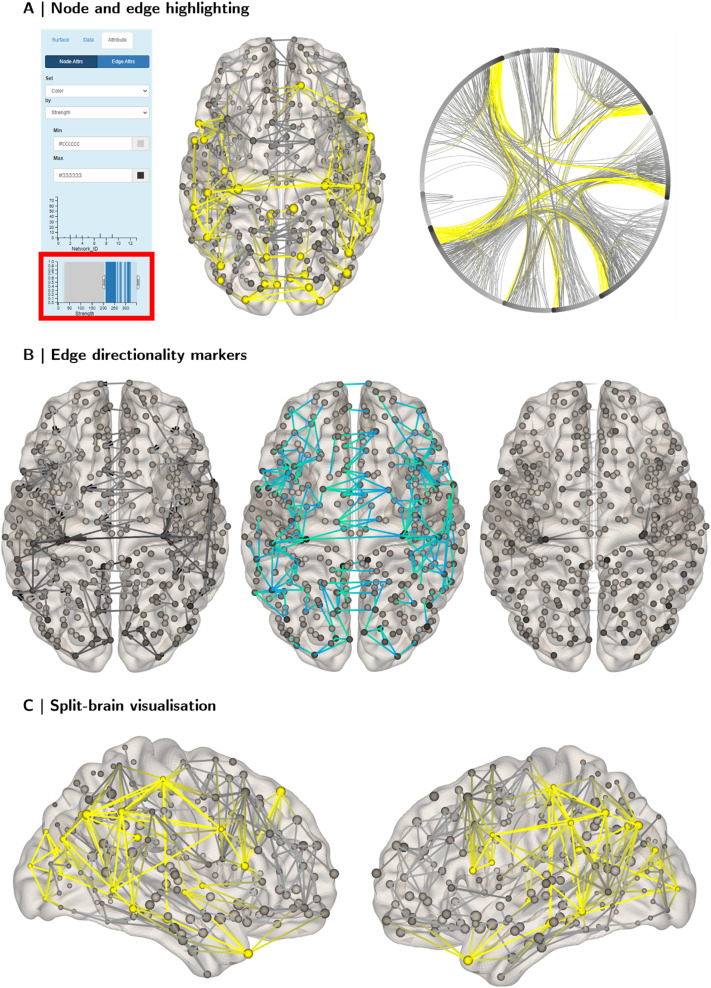
Additional functionality available in 3D visualizations. (A) Node and edge highlighting by selecting the range of an attribute. Left: selection panel showing selection of the nodes with the highest strength. The user can select a range by clicking and dragging the histogram of the desired attribute (red box). Right: the nodes that satisfy that range are highlighted yellow in the 3D visualization, as are the edges that end at those nodes (in both the 3D and 2D visualization). The color of the nodes shows the nodes’ strength. Note that in the 2D visualization, nodes are bundled according to their 12-network allocation in the HCP Cole-Anticevic scheme (Ji et al., 2019). (B) Edge display options for directed graphs. Left: arrow mode places an arrowhead indicating the edge direction. Middle: gradient mode colors the edges from the user-specified start to the end color. Right: transparency mode displays edges with linearly increasing opacity from the start to the end. See Supporting Information for an example of animation mode, which moves a red bar from the start to the end along each edge. (C) The split view button (M) will show the left and right hemispheres separately. Note that interhemisphere connections will not be shown in this view. The data and visualizations in this figure can also be viewed interactively at https://immersive.erc.monash.edu/neuromarvl/?save=b3ebc1c7-6776-44c0-8844-772bfd9e042e_49.127.37.54.

For directed networks, the following options are provided for visualizing edge directionality (Figure 3B): *arrow*, where arrowheads are placed on edges; *gradient*, which linearly transitions the edge color from start to end; *transparency*, which linearly increases opacity of the edge from start to end; and *animation*, which moves red bars along the edges from start to end (see https://immersive.erc.monash.edu/neuromarvl/?save=bae0b5bd-941b-424c-9f20-43892a097c48_49.127.39.199).

#### Display panel controls.

The Display panel itself contains additional controls for interactively modifying the network visualization. The top-right corner presents several buttons for generating distinct visualizations. Specifically, they include the following options:

1. Orientation selection, which allows users to snap the brain surface to fixed top (T), bottom (B), left (L), right (R), front (F), and back (P) orientations. The user can also click and drag with the right mouse button to select an arbitrary surface orientation.
2. Split view (M; Figure 1*), in which users can split the surface into the left and right hemispheres (Figure 3C) and prevent plotting of interhemispheric connections in the 3D plot.
3. Anticlockwise auto-rotation (Figure 1†), which causes the primary view to rotate in an anticlockwise fashion according to the current view.

The bottom-left corner of the display panel presents a slider that can be used to interactively adjust the number of edges that are displayed. For performance, a maximum of 1,000 edges can be viewed at any one time.

Next to the slider, several buttons can be used to control the properties of a secondary, nonanatomical (i.e., topological or circular) projection. These are explained in the following sections.

### Nonanatomical Projections

NeuroMArVL provides options for topological and circular projections of the connectome. These projections supplement and are yoked to the main anatomical 3D view, allowing users to visualize corresponding node and edge features in both views (Figure 1B) and easily move between different projections. With hundreds of edges being visualized at a time, it may be difficult to see the edges originating at a given node; yoking allows a node to be highlighted in the 3D visualization to reveal its corresponding edges in a concurrently display nonanatomical projection (or vice versa).

#### Topological (graph) projections.

Topological projections can be generated either in 2D or 3D and display nodes and edges in a way that corresponds to their topological relations. They can be particularly useful for understanding how communities are situated with respect to each other or for identifying topologically central nodes. The topological projections utilize Cytoscape.js (Shannon et al., 2003) to alter the connectome positions and organize the nodes into distinct clusters according to a selected bundling attribute. The bundling attribute is selected from the available discrete or mask-valued node attributes.

**Figure 4.**
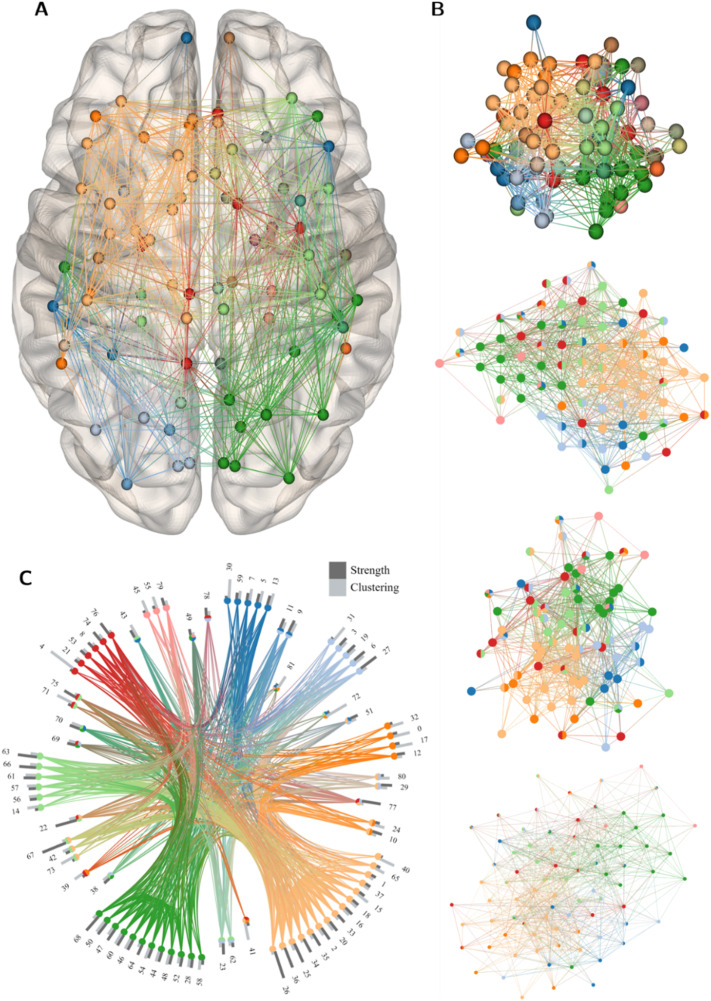
Examples of alternate visualizations that can accompany the primary 3D anatomical visualization. (A) Example network shown in MNI space. The nodes and edges are colored according to module assignment, with nodes potentially assigned to multiple modules. (B) Example of alternative graph embeddings. First, 3D topological projection; second, using the CoLa algorithm; third, using the COSE-bilkent algorithm; and fourth, using the COSE algorithm. (C) Example circular projection of the same network. Nodes allocated to exactly one module are shown in the outermost ring, and nodes allocated to more modules are shown in progressively smaller rings. Nodes belonging to multiple modules (e.g., Nodes 43, 49, and 81) contain colors of each module as a pie chart. Also note the bar chart embedded around the chord diagram, which shows the strength and clustering coefficient of each node. The data and visualizations in this figure can also be viewed interactively at https://immersive.erc.monash.edu/neuromarvl/?example=40496078-effa-4ac3-9d3e-cb7f946e7dd1_137.147.133.145.

NeuroMArVL provides six main topological projections:

- CoLa 3D and CoLa 2D (Figure 4B, first and second): a constraint-based layout algorithm that extends force-directed visualizations by adding separation constraints, which enforce a minimum distance between pairs of nodes (Dwyer et al., 2006).
- COSE (Figure 4B, third): The COSE layout algorithm is a physics-based method that produces fast results for large, complex graphs (Dogrusoz et al., 2009).
- COSE-bilkent (Figure 4B, fourth): a modification of the COSE algorithm that is optimized for larger graphs, and performs multilevel nesting of nodes into clusters (Dogrusoz et al., 2009).
- Grid and Concentric: These options order the nodes by module, and then arrange all the nodes in a square grid/circle such that modules remain grouped together.

#### Circular diagrams.

Circular projections position nodes around the perimeter of a circle and depict edges with connecting arcs (Figure 4B). Node positions can thus be varied and/or bundled to reveal different distinct aspects of network organization, such as community structure, that are not readily appreciated in the anatomical projection.

NeuroMArVL supports two levels of node reordering based on attributes entered by the user: (a) the bundling of nodes into high-level clusters, such as distinct communities (see Figure 1 for an example), and (b) the sorting of nodes within these bundles, for example, by node strength (Figure 3A). When sorted based on these attributes, intramodule edges have a short arc through the circle, while intermodule edges bisect the diagram. Other features can also be tailored, such as node coloring (e.g., to distinguish communities) or the inclusion of simple bar charts at each node location to illustrate variations in some node attribute (e.g., node degree). Multiple such bars can be displayed, and their color is fully customizable. For example, in Figure 3A, nodes are first bundled by their community affiliation based on a standard network parcellation (Ji et al., 2019). Within each bundle, they are sorted by node strength (the sum of edge weights terminating at that node). When combined with the highlighting feature of the viewer, Figure 3A shows that any given high-strength node is connected strongly to other high-strength nodes. This suggests the presence of a “rich club” of highly connected nodes, a feature that is known to exist in the connectomes of diverse species (Arnatkeviciute et al., 2021; Arnatkevic̆iūtė et al., 2018; de Reus & van den Heuvel, 2013; Fulcher & Fornito, 2016; Towlson et al., 2013; van den Heuvel et al., 2016; van den Heuvel & Sporns, 2011).

Figure 4C shows an example of a circular diagram for a network decomposed into eight overlapping communities (i.e., individual nodes can belong to more than one community). NeuroMArVL identifies nodes belonging to more than one community by positioning them along concentric rings closer to the center of the circle, with each level identifying nodes that belong to an increasing number of communities.

### Reproducibility and Data Sharing

NeuroMArVL supports various features, accessible through the data tab, that facilitate the generation of reproducible visualizations by both an individual user and multiple users working collaboratively. This is done by saving and sharing visualization settings, which refer to the appearance of the brain surface, the nodes and edges in the primary view, and the appearance of the secondary view. If a surface model was uploaded, this will also be saved.

The “Save settings to file” function downloads the current settings to a local JSON file. This file can be manually edited if desired. It can later be uploaded using the “Load settings from file” function, which applies these saved settings to the current visualization. This functionality does not save any data to remote servers and the files remain entirely in the control of the user. The JSON file can act as a settings template that can produce reproducible visualizations across multiple datasets: These datasets can use different attributes and adjacency matrices, but must have the same dimensions (vertices and edges) and node labels. This functionality is all maintained when NeuroMArVL is built and deployed locally.

The “Save settings on webserver and share link” function saves the settings and the coordinates, matrix, attributes and labels on the webserver permanently (see Saved Data). Each data file is saved as is on the web server using randomly generated file names, and a link that allows users to share their visualizations with other researchers is generated. Any uploaded data may be deleted on request.

**Figure 5.**
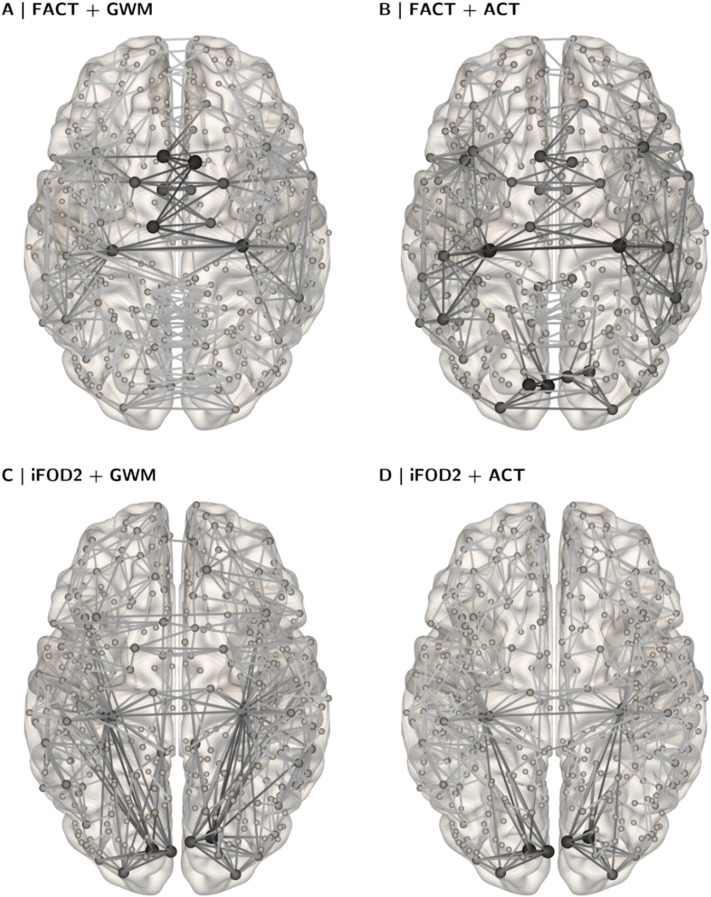
Visualizations generated programmatically in batch mode. Structural connectomes from four different tractography algorithms are shown: (A) deterministic tractography with a gray matter-white matter mask; (B) deterministic tractography with nonanatomical streamlines removed; (C) probabilistic tractography with a gray matter-white matter mask; (D) probabilistic tractography with nonanatomical streamlines removed. Nodes are colored by strength (darker = stronger) and edges are colored by node. FACT, fiber assignment by continuous tractography; iFOD2, second-order integration of fiber orientation distributions; GWM, gray matter-white matter mask; ACT, anatomically constrained tractography. The data and visualizations in this figure can also be viewed interactively at https://immersive.erc.monash.edu/neuromarvl/?save=1d6b6ad1-1951-4607-bbd7-48dda276244b_118.138.254.226.

NeuroMArVL also provides functionality to visualize and export images for multiple adjacency matrix/attribute pairs automatically using the “Batch mode” function (Figure 2A, second panel). Firstly, the user sets up a visualization in one dataset by choosing a brain surface model, node locations/labels, and any desired 2D projection, placing and orienting the elements in their appropriate location. Then, in the batch mode tab, the user selects pairs of adjacency/weight matrix and attribute files for which visualization is required. These new adjacency and attribute files must have the same dimensions and format as the originals. After selecting the format and size of the output image files, NeuroMArVL iterates through all the selected adjacency/weight matrix and attribute file pairs and generates images with the same visualization parameters as the original, which can be saved to the local hard drive. As a use case, Figure 5 depicts the images generated from batch mode showing differing edge and node strengths across preprocessing methods for diffusion-based connectomes for a common parcellation scheme.

### Demonstrating NeuroMArVL’s Features

NeuroMArVL has already been used to generate visualizations for various types of similarity measures between brain regions (see, e.g., Figures 4 and 5 in Liu et al. (2024); Figure 2 in Imms et al. (2021); Figure 3 in Fulcher and Fornito (2016); and Figures 2, 3, and 4 in Hanekamp and Simonyan (2020). Here, we demonstrate how the aforementioned features can be used in typical neuroimaging workflows to aid data analysis.

Figure 5 shows an example of the figures generated by batch mode, in which multiple figures are programmatically generated according to a common set of parameters. In this example, we compared structural connectomes from a variety of diffusion MRI tractography pipelines (Gajwani et al., 2023; Oldham et al., 2020). Previous analyses using these data focused on node degree distributions, a scalar value attributed to each node (Bullmore et al., 2009). Node degree can be easily visualized on the cortical surface using typical programming languages or visualization libraries, as can most other node-level attributes. However, NeuroMArVL allows for comparison of a larger suite of graph theoretic features, including both node-level and edge-level attributes (Figure 5). The visualizations clearly display the difference in interhemispheric connections between deterministic tractography (Figure 5A–B) and probabilistic tractography (Figure 5C–D). Similarly, the distribution and connectivity of the “hub” nodes (the nodes with the greatest strength) change significantly when anatomically constrained tractography (Smith et al., 2012), a processing step that filters anatomical implausible diffusion tractography streamlines, is used (Figure 5A–B).

**Figure 6.**
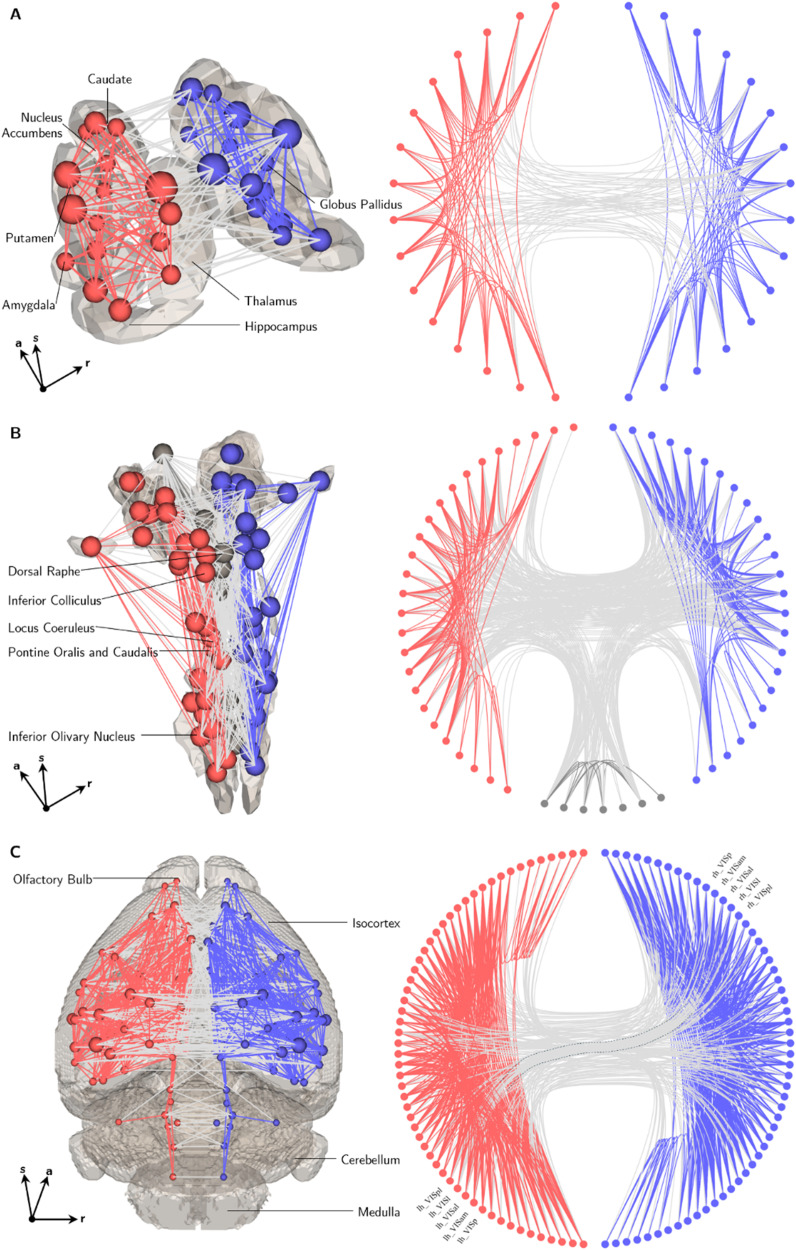
Example visualizations with data other than those from human cortex. NeuroMArVL can visualize connectomes from a variety of sources, including human subcortex (A), human brainstem (B), and mouse whole brain (C). In each visualization, the left hemisphere is shown in red and right hemisphere in blue, with interhemispheric connections shown in light gray. Note the medial structures shown in dark gray in (B), and the strong connection between lateral visual areas in (C). The data and visualizations in this figure can also be viewed interactively at https://immersive.erc.monash.edu/neuromarvl/?save=11d02130-e791-4b41-9a05-e3eaf87ed6cf_49.127.37.54, https://immersive.erc.monash.edu/neuromarvl/?save=92d4fa6a-2f65-443c-89a9-6d68b8fd245d_49.127.37.54, and https://immersive.erc.monash.edu/neuromarvl/?save=56af84bf-6cd1-4870-b859-d66ffca96af6_49.127.51.226.

NeuroMArVL is not limited to connectomes of the human cerebral cortex. Figure 6 shows examples of the human subcortex, the human brainstem, and the mouse whole brain. Brain surfaces and node locations must be in register (e.g., the mouse surface and vertex locations are all registered to the Allen CCF v3), but NeuroMArVL is otherwise space- and scale-agnostic. NeuroMArVL is also agnostic to the type of information used to calculate the similarity between network nodes since adjacency matrices and attributes must be user specified. For instance, Figure 6A shows subcortical SC derived from diffusion MRI tractography, while Figure 6B shows brainstem FC derived from BOLD functional MRI. Figure 6C shows a mouse connectome mapped using viral tract tracing (for interactive visualizations, see https://github.com/NSBLab/neuromarvl/blob/master/example_data/README.md). Despite the disparate methods used to map brain connectivity, the same visualization tools can be used to reveal key structural properties of the networks.

## DISCUSSION

NeuroMArVL was developed as an open-source, online tool for connectome viewing and figure generation. It can generate interactive, yoked visualizations without the need for users to have any programming experience. The JavaScript frameworks upon which the software rely allow the viewer to be more interactive than most existing tools. They also permit viewing and generating figures in environments where is not possible to install new software (e.g., on enterprise installations). The combination of the anatomical, circular, and topological visualizations allows researchers to combine multiple approaches to analyzing connectomes, facilitating novel understanding of connectome properties. Users are also able to contribute new features to the open-source data repository that is associated with the viewer. Finally, the ability to generate shareable links for visualizations, in addition to the saving and batching functions, support reproducible figure generation pipelines for both individual users and collaborative teams, in addition to a more detailed, interactive interrogation of findings reported in published manuscripts.

One of the key motivations behind NeuroMArVL is the ability to generate reproducible, shareable visualizations. Computational neuroscience has been recently plagued by a “reproducibility crisis” (Miłkowski et al., 2018), in part due to difficulties comparing processing pipelines, data, and results. NeuroMArVL aims to increase the reliability of results in the field by improving current practices in two ways. First, batch mode enables easy visualization and comparison of the results of different processing pipelines. Users can generate connectomes from a variety of pipelines, import these into NeuroMArVL using the batch mode feature, and export the visualizations in a few simple steps. This will allow users to easily confirm that their results are robust to different connectome processing choices, which is important given evidence that such processing choices can affect many network properties (Ciric et al., 2018; Gajwani et al., 2023; Oldham et al., 2020; Parkes et al., 2018). Secondly, the use of shareable links allows reviewers and readers to interact with other researchers’ connectome data quickly and easily. Presently, data is normally shared in one of two ways: (a) as pregenerated figures, in which only the data that have already been presented can be viewed; or (b) raw data, which reviewers/readers must reformat and sometimes reprocess before generating their own visualizations. NeuroMArVL’s shareable links now allow reviewers/readers to have fully interactive visualizations of the data within a manuscript, allowing for further rigor when reading manuscripts. Together, we hope these features will help increase the reproducibility of results in the field.

Future directions for NeuroMArVL may see it work more closely with other visualization tools in the field, for example, Schol-AR (Ard et al., 2022). Schol-AR is an augmented reality tool that superimposes plots, data, and animations onto digital articles. Schol-AR visualizations can be viewed via a desktop setup (e.g., when reading a PDF on a laptop), or via a smartphone app (e.g., when perusing posters at a conference). A future direction for NeuroMArVL could be to integrate the interactive 3D plots into Schol-AR. This would further streamline the viewing and sharing of NeuroMArVL’s interactive visualizations.

Future work may also see NeuroMArVL used to visualize any network where the nodes can be embedded in Euclidean space (≤3D). This could include cellular level imaging, transportation maps (e.g., London train map [Luppi et al., 2024]), or high-dimensional data that have undergone dimensionality reduction using algorithms such as t-Distributed Stochastic Neighbor Embedding (tSNE), Uniform Manifold Approximation and Projection (UMAP), or multidimensional scaling (MDS).

### Limitations

NeuroMArVL remains an open-source project that welcomes community contributions. We hope that these contributions will help address some of the current limitations in the software. The primary limitation is that NeuroMArVL requires a Chrome or Chromium-based browser to work. This includes most common browsers including Chrome, Edge, Opera, Brave, and Vivaldi, but does not include Firefox or Safari due to display issues that prevent figure export. Future updates or compatibility patches from the makers of these browsers may see this issue resolved.

NeuroMArVL currently visualizes connections as the straight-line connection between node locations in 3D space. However, anatomical connections in the brain follow curved paths travelling along defined tracts. Future work could allow users to incorporate streamline anatomical information into the viewed, allowing connections to be plotted along these anatomical pathways.

NeuroMArVL allows for the separation of brain hemispheres, allowing users to view the medial aspect of deep structures such as those of the subcortex. By assigning a mask or a Boolean attribute to subcortical structures, users can also visualize these structures in a separate color or view. However, NeuroMArVL does not currently permit other segregated visualizations of the cortex vs subcortex. Future work may consider allowing users to generate two visualizations (e.g., two circular projections), one for cortical structures and one for subcortical structures.

### Conclusions

Here, we have presented NeuroMArVL, a software package for interactive and reproducible connectome visualization sharing. It is available as a hosted online resource at https://immersive.erc.monash.edu/NeuroMArVL/. It is an open-source tool available to the community, with source code available for download via GitHub at https://github.com/NSBLab/NeuroMArVL. We welcome community contribution and hope that novel visualizations of brain networks will allow researchers to better understand brain networks in humans and other species.

## ACKNOWLEDGMENTS

The authors would like to thank Thanh Nhan Pham, Mingzheng Shi, and Nicholas Smith for their contribution to the development of the package. This work was supported by the use of the Australian Research Data Commons (ARDC) Nectar Research Cloud, a collaborative Australian research platform supported by the National Collaborative Research Infrastructure Strategy (NCRIS)-funded ARDC. This work was supported by Monash eResearch capabilities, including M3.

This work was supported by the National Health and Medical Research Council (IDs: 1197431, 1146292, 1136192), Australian Research Council (IDs: FL220100184, FT130100589, and DP200103509), the Sylvia and Charles Viertel Charitable Foundation, and an Australian Government Research Training Program Scholarship.

## AUTHOR CONTRIBUTIONS

Christopher Leslie Adamson: Software; Writing – original draft; Writing – review & editing. Mehul Gajwani: Data curation; Software; Writing – review & editing. Matthias Klapperstueck: Project administration. James Manley: Conceptualization; Software. Tim Dwyer: Conceptualization; Investigation; Software. Alex Fornito: Conceptualization; Funding acquisition; Investigation.

## FUNDING INFORMATION

Alex Fornito, National Health and Medical Research Council (https://dx.doi.org/10.13039/501100000925), Award ID: 1197431. Alex Fornito, National Health and Medical Research Council (https://dx.doi.org/10.13039/501100000925), Award ID: 1146292. Alex Fornito, National Health and Medical Research Council (https://dx.doi.org/10.13039/501100000925), Award ID: 1136192. Alex Fornito, Australian Research Council (https://dx.doi.org/10.13039/501100000923), Award ID: FL220100184. Alex Fornito, Australian Research Council (https://dx.doi.org/10.13039/501100000923), Award ID: FT130100589. Alex Fornito, Australian Research Council (https://dx.doi.org/10.13039/501100000923), Award ID: DP200103509. Sylvia and Charles Viertel Charitable Foundation (https://dx.doi.org/10.13039/100008717).

## CODE/DATA AVAILABILITY AND CONTRIBUTIONS

All code is available via the repository at https://github.com/NSBLab/neuromarvl. NeuroMArVL can be downloaded, built, and used offline if desired. Contributions can be made and reviewed using GitHub’s forking and pull requests features.

The data included in the viewer are available via the website (https://immersive.erc.monash.edu/neuromarvl/brain-app/data/example.zip) and via the repository. Users can also contribute data by contacting the authors. Surface models must be in MNI space and would be made available in the selectable list. Parcellation schemes, comprising of node locations and labels, would be made available for download via GitHub; these could then be uploaded via the data tab in the NeuroMARvL interface

## Note

^1^ Yoked visualizations are those where multiple panels have the same viewing parameters, rotation/zooming/panning, and selections applied.

## TECHNICAL TERMS

Topological properties (of connectomes): Quantitative descriptions of graph structure, taken from graph theory and applied to brain connectomes.
Yoked (visualizations): Connect properties of multiple visualizations so changes/events occurring on one are propagated to all others.
Connectome: A graph-based representation of relationships between regions of interest (typically) in the brain.
Widget (webpage): Interactive objects on the webpage, for example, buttons, list boxes, and radio buttons.
Module (graph): A subset of nodes that have high mutual connectivity and low connectivity to the rest of the graph.
Parcellation: A subdivision of the cerebral cortex into regions.
UUID: Universal Unique IDentifier, a large hexadecimal number, used in this software to generate unique, random file names.
Centrality (graph): The proportion of shortest paths a node belongs to. High-centrality nodes can be thought of as the “trunk” of the connectome, while low-centrality nodes are the “leaves.”
